# Hypokalemic paralysis in an adolescent following dexamethasone and B12 injection: A case report and literature review

**DOI:** 10.1016/j.heliyon.2025.e41675

**Published:** 2025-01-09

**Authors:** Keivan Sahebi, Hassan Foroozand, Mohammad Bahmei, Raziee Taghizadeh, Samane Zare, Soroor Inaloo

**Affiliations:** a Student Research Committee, School of Medicine, Shiraz University of Medicine, Shiraz, Iran; b Pediatric Neurology Department, Nemazee Hospital, Shiraz, Iran; c Neonatal Research Center, Shiraz University of Medical Sciences, Shiraz, Iran

**Keywords:** Hypokalemia, Paralysis, Dexamethasone, Vitamin B12, Case report, Glucocorticoid-induced hypokalemia, Neurobion, Adolescent case study

## Abstract

The widespread use of glucocorticoids in clinical practice may occasionally be complicated by hypokalemic paralysis. Previously, only a few cases of glucocorticoid-induced hypokalemic paralysis in healthy adults had been reported. Intriguingly, cases of B12-induced hypokalemia have previously been reported in patients with pernicious anemia. Recently, we experienced a case of hypokalemic paralysis in an adolescent following intramuscular injections of dexamethasone and vitamin B12. Upon exclusion of other causes, a presumptive diagnosis of glucocorticoid-induced hypokalemic paralysis, with a possible but uncertain contribution from B12 supplementation, was made for the patient. After potassium replacement therapy, the patient fully recovered and was discharged after five days. Although glucocorticoids are known to cause hypokalemia through mechanisms such as enhanced transcellular shift or renal excretion, the potential role of vitamin B12 in worsening this effect remains unclear. It is hypothesized that B12 supplementation under certain conditions could influence electrolyte balance and potentially amplify the hypokalemic effects of glucocorticoids. However, this hypothesis is based on a few cases, and further pathophysiological and clinical studies are needed to clarify whether B12 supplementation exacerbates hypokalemia induced by glucocorticoids or if the observation is coincidental or context-specific. Meanwhile, clinicians should be cautious when prescribing glucocorticoids, particularly in combination with B12 supplements. This includes ensuring that both are administered only when clinically indicated, monitoring vital signs and serum potassium levels in at-risk patients, and considering alternatives when appropriate.

## 1 Introduction

Glucocorticoids are a widely used group of drugs prescribed for various medical conditions, such as allergic reactions, autoimmune diseases, and organ transplant rejection [1]. Commonly prescribed glucocorticoids include dexamethasone, hydrocortisone, prednisolone, and methylprednisolone. These drugs are primarily metabolized by the liver and excreted *via* the kidneys [2]. Hypokalemic paralysis is a rare but significant adverse effect of glucocorticoids [[3], [4], [5], [6]]. While the precise mechanisms remain unclear, several hypotheses have been proposed to explain glucocorticoid-induced hypokalemia. These compounds may indirectly activate sodium/potassium adenosine triphosphatase (Na^+^/K^+^-ATPase), facilitating the intracellular transport of potassium [4,7,8]. Glucocorticoids may also promote renal K^+^ excretion through their mineralocorticoid-like effects [9]. Additionally, a well-known adverse effect of glucocorticoids is the induction of hyperglycemia and insulin resistance. This leads to compensatory hyperinsulinemia, which enhances the K^+^ shift into cells, thereby exacerbating hypokalemia [10].

According to previous reports, hypokalemic paralysis has been reported to be induced by glucocorticoids used in different clinical situations, such as Graves’ disease, muscle cramps, or dermatologic complaints [[11], [12], [13]]. Ebrahimi et al. reported a 34-year-old male who presented with quadriplegia following an intramuscular (IM) injection of 4 mg betamethasone due to allergic symptoms (rhinorrhea, itchy eyes, and pruritus on his face) [11]. On arrival, he was conscious and responsive, but unable to ambulate. On the Medical Research Council (MRC) scale, muscle power was 1/5 bilaterally in the lower extremities and 3/5 in the upper limbs. Additionally, hypotonia, diminished deep tendon reflexes (DTRs), and bilateral flexor plantar responses were observed. Other neurologic examinations, including cranial nerves, sensory function, and sphincter function, were intact. The symptoms resolved completely after intravenous (IV) K^+^ treatment, and the patient was discharged with a final diagnosis of HPP induced by glucocorticoids. Another reported case involved a 26-year-old man without any relevant past medical history who was admitted with acute bilateral lower limb weakness [12]. The patient had an IM injection of 5 mg dexamethasone for his shoulder pain one day prior to symptom onset. Laboratory tests revealed severe hypokalemia (K^+^ = 1.7 mEq/L). After oral and IV K^+^ supplementation, hypokalemia resolved, and muscle strength returned to normal (serum K^+^ = 4.0 mEq/L), and the patient was discharged without any complications.

Patients with thyrotoxic periodic paralysis (TPP) represent an important subset of those who develop hypokalemic paralysis following glucocorticoid administration. For instance, a new case of TPP was identified in a patient presenting with severe hypokalemia following an epidural steroid injection [13]. The patient was a 36-year-old man who received an epidural injection of an unknown steroid 4 h prior to his admission. On examination, he had lower extremity weakness (1/5) and diffuse hyporeflexia. Except for a history of recent weight loss and an enlarged thyroid on examination, there were no other notable signs and symptoms suggestive of hyperthyroidism, such as ophthalmopathy, tremors, or heat intolerance. The diagnosis of TPP was confirmed by TSH of 0.1 μIU/ml and elevated serum anti-thyroglobulin, anti-thyroid peroxidase, and anti-TSH receptor antibodies. K^+^ levels were corrected by administering IV K^+^ replacement, and the patient was discharged with propranolol to manage symptoms of hyperthyroidism.

Additionally, vitamin B12 supplements may be used in several clinical conditions, even in the absence of documented B12 deficiency [14]. Nonetheless, to date, clinical B12-induced hypokalemia has been reported primarily in cases of megaloblastic anemia [15,16], possibly *via* K^+^ shift into the newly synthesized erythrocytes. As a result, based on current reports, it is postulated that concurrent administration of glucocorticoid and vitamin B12 may, at least in susceptible patients, exacerbate hypokalemia, though additional studies are necessary to fully investigate this hypothesis and its clinical relevance. Clinicians should prescribe both glucocorticoids and vitamin B12 supplementation judiciously and only when clinically warranted. Moreover, patient education and close follow-up are crucial for those receiving glucocorticoids and vitamin B12. Here, we report a case of hypokalemic paralysis in a previously healthy boy who presented with progressive ascending quadriparesis after IM administration of dexamethasone and Neurobion™ (a multivitamin preparation containing vitamins B1, B6, and B12). This study also provides a review of the literature on hypokalemic paralysis following glucocorticoid administration.

## 2 Case report

A 17-year-old boy was brought to the emergency department (ED) presenting with acute weakness in both the upper and lower extremities, which began 5 h before admission. The patient had no clinically significant past medical history and was otherwise healthy. Over the past two days, he experienced upper respiratory infection (URI) symptoms, such as fever, sneezing, cough, rhinorrhea, and nasal congestion. The patient visited a local general practitioner for his URI symptoms, and the clinician prescribed an IM injection of 8 mg of dexamethasone and vitamins B1, B6, and B12 (Neurobion™ Merck, containing 100 mg thiamine chloride hydrochloride, 100 mg pyridoxine hydrochloride, and 1 mg cyanocobalamin in a 3 mL aqueous solution) for him. The clinical indication for these injections is unclear but appears to have been aimed at attenuating URI symptoms (dexamethasone) and the patient's generalized fatigue (Neurobion™). After 24 hours, the patient developed progressive and ascending weakness in the lower extremities. On admission, the patient was febrile (37.8 °C), hemodynamically stable, and showed no signs of respiratory distress. The physical examination revealed muscle power of 2/5 in both upper and lower extremities and hyperreflexia (3^+^/4^+^) in distal and proximal DTRs, with no sensory abnormality. Moreover, the patient was unable to support his neck. The rest of the physical examination was unremarkable.

The initial biochemistry laboratory data revealed serum K^+^ and Na^+^ levels of 2.3 and 139 mEq/L, respectively. Brain computed tomography (CT) scan and magnetic resonance imaging (MRI) showed no evidence of intracerebral pathologies or infarctions. Table 1 summarizes the clinical course, treatments, and laboratory results of hematological and biochemical tests. A presumptive diagnosis of hypokalemic paralysis was made, and the patient was transferred to the intensive care unit (ICU). Normal serum creatine phosphokinase (CPK) and lactate dehydrogenase (LDH) levels ruled out myopathies. Urine toxicology was performed to rule out toxicities from substances of abuse (Table 2). Lower extremity radiographs also ruled out orthopedic causes of pseudo-paralysis, such as fractures. Chest X-ray (CXR), urinalysis, urine culture, and blood cultures were obtained to identify a potential infection site, but all results were negative. After 24 hours of potassium chloride (KCl) replacement, serum K^+^ levels reached 4.3 mEq/L, accompanied by an improvement in muscle strength. A comprehensive history was obtained from the patient and his parents. They reported that the patient had not used any medications other than the two injections described earlier and had never experienced a similar episode. He also had no history of gastrointestinal (GI) symptoms, such as diarrhea or vomiting, in the past few days. The rapid improvement, with full recovery of muscle strength by day 3, allowed the patient to be transferred to the ward and subsequently discharged on day 5 with a diagnosis of HPP of non-familial type. However, genetic testing was not performed due to the patient's financial limitation and unwillingness. On one-week follow-up, serum electrolytes were within the normal ranges (Na^+^ = 140 mEq/L and K^+^ = 4.4 mEq/L), with serum vitamin B12 and folic acid of 344 pg/ml and 8.95 ng/ml, respectively. Serum homocysteine was 6.8 μmol/L, and serum and urine methylmalonic acid were 118 nmol/L and 1.2 mmol/molar, respectively.

**Table 1:** The clinical course, treatment, and results of hematological and biochemical tests.

	**Day 1**	**Day 2**	**Day 3**	**Day 4**	**Day 5**	**1 week F/U**
**Muscle power (MRC scale)**	2/5	3/5	4/5	4/5	5/5	
**Treatment (24 h IV KCl** [mEq/L]**)**	60	20	20	–	–	
**CBC**						
WBC ( × 10^3/μL)	24.9	20.8			7.5	
RBC ( × 10^6/μL)	5.35	5.00			5.36	
Hb (g/dL)	16.3	15.5			16.4	
Hct (%)	44.1	41.6			45.4	
MCV (fL)	82.4	83.2			84.7	
MCH (pg)	30.5	31.0			30.6	
MCHC (g/dL)	37.0	37.3			36.1	
Platelets (x10^3/μL)	260	207			181	
RDW (%)	13.2	13.3			12.7	
PDW (fL)	14.6	14.7			13.7	
MPV (fL)	11.1	11.3			10.6	
P-LCR (%)	34.5	35.2			30.1	
Band (%)		1			1	
Neutrophil (%)		84			54	
Lymphocyte (%)		9			30	
Monocyte (%)		6			4	
Eosinophil (%)		–			10	
Basophil (%)		–			1	
**Biochemistry**						
BS (mg/dL)	118					
BUN (mg/dL)	14		12			
Cr (mg/dL)	1.03		0.98			
Ca (mg/dL)	10.3		9.6			
Na (mEq/L)	139	139	138	137	140	140
K (mEq/L)	2.3	4.3	4.5	4.6	4.5	4.4
Mg (mg/dL)	1.9					
Phosphorus (mg/dL)		2.7	4			
Total protein (g/dL)		6.8				
Alb (g/dL)		4.1				
Glob (g/dL)		2.7				
AST (U/L)		27				
ALT (U/L)		12				
T.B (mg/dL)		0.80				
D.B (mg/dL)		0.40				
Alk-p (U/L)		171				
**ESR (mm/h)**		8				
**CRP (mg/L)**		19

**Table 2:** The laboratory results of urine analysis and toxicology.

**Urine analysis**	
Color	Yellow
Appearance	Clear
PH	alkaline
Specific gravity	1.006
Protein	Negative
Blood/Hb	Negative
Glucose	Negative
Ascorbic Acid	Negative
Urobilinogen	Negative
Bilirubin	Negative
Nitrite	Negative
Ketone	Negative
Leukocytes esterase	Negative
R.B.C (cell/HPF)	0–1
W.B.C (cell/HPF)	0–1
Epithelial cell (cell/HPF)	0–1
**Urine Toxicology**	
Cocaine	Negative
Amphetamine	Negative
Methamphetamine	Negative
Marijuana	Negative
Methadone	Negative
Morphine	Negative
Benzodiazepines	Negative
Tricyclic antidepressant	Negative
Buprenorphine	Negative

## 3 Discussion

Hypokalemia can occur due to three main causes: (i) reduced K^+^ intake, (ii) increased K^+^ loss through the GI tract and renal excretion, or (iii) K^+^ intracellular shift [17]. One genetic cause of hypokalemia is hypokalemic periodic paralysis (HPP). Mutations in genes encoding the subunit of L-type voltage-dependent calcium channel Cav1.1 (CACNA1S) and skeletal muscle sodium voltage-gated channel Nav1.4 (SCN4A) are responsible for channelopathies observed in HPP [18,19]. Paralytic episodes associated with HPP may be triggered by factors such as carbohydrate-rich meals, stress, exercise, or certain medications.

Despite their widespread therapeutic use, glucocorticoids may cause hypokalemic paralysis, either directly or in individuals with a genetic predisposition [4,6,[11], [12], [13],[20], [21], [22]]. A 26-year-old healthy man developed acute lower limb weakness one day after receiving an IM injection of 5 mg dexamethasone in his right shoulder, which was administered to treat previous muscle cramps [12]. On admission, the patient was febrile (38.1 °C) and exhibited reduced muscle power of the bilateral proximal lower limbs (1/5). In laboratory investigations, he had mild leukocytosis (10.8 × 10^3^/μL) and severe hypokalemia (K = 1.7 mEq/L). After a total of 120 mEq oral and IV potassium supplementations, hypokalemia was corrected (K^+^ = 4.0 mEq/L) and muscle power restored. Another case involved a 32-year-old man with acute lower and upper limb weakness [20]. The symptoms developed one day after he had a dexamethasone injection for an ophthalmological procedure (retinal detachment). Neurological examination revealed reduced muscle power in the lower and upper limbs (2/5), while DTRs and sensation were intact. Serum K^+^ was initially 2.38 mEq/L. After oral and IV K^+^ replacements, muscle power was improved, and the patient was discharged with the final diagnosis of HPP of the non-familial type. Additionally, two cases of hypokalemic paralysis in healthy adults—a 42-year-old man and a 34-year-old man—were reported after low-dose glucocorticoid administration for urticaria [6]. The administration routes were IM and IV, respectively. In both cases, an acute muscle weakness occurred within hours of glucocorticoid administration and rapidly resolved after K^+^ replacement. Another case report described a 26-year-old man who presented with similar symptoms of weakness in the left arm and leg. Six hours before the onset of symptoms, he received an 8-mg IM dexamethasone injection. Following IV K^+^ replacement, serum K^+^ levels normalized, and muscular weakness resolved [3].

In addition to their role in triggering HPP, glucocorticoids can induce hypokalemia through several mechanisms. Glucocorticoids can enhance intracellular K^+^ shifts by activating the Na^+^/K^+^-ATPase [7,8]. In a patient with HPP, this initial K^+^ influx into cells causes a paradoxical depolarization in the skeletal muscle cell membrane, leading to inactivation of Na^+^ channels and loss of muscle excitability [18,23]. Furthermore, studies have shown that glucocorticoids may enhance Na^+^ reabsorption in the renal tubules and increase renal K^+^ excretion in the aldosterone-sensitive distal tubules [24]. Another mechanism of glucocorticoid-induced hypokalemia is the induction of hyperglycemia, which leads to hyperinsulinemia. This hyperinsulinemia shifts K^+^ ions into the cells and exacerbates hypokalemia [25].

B12 supplementation has previously been recognized as a transient cause of hypokalemia in patients with megaloblastic anemia due to increased cell proliferation and intracellular K^+^ shift [15]. The present study reports an adolescent with hypokalemic paralysis following an IM injection of both dexamethasone and vitamin B12 (Neurobion). This raised the hypothesis that the redistribution of K^+^ by glucocorticoids may be further exacerbated when B12 supplements are concurrently administered. However, pathophysiological studies are needed to determine whether B12 supplementation in non-anemic patients is a risk factor for exacerbating glucocorticoid-induced hypokalemia.

Our patient is the first adolescent case with concurrent glucocorticoid and B12 injections. Recently, two similar adult cases were also reported by Chen et al. [21]. The patients were two brothers, 32- and 38-year-old, who presented to the ED with numbness and arms and legs weakness 16 h following unknown gluteal IM injections, which were suspected to contain vitamins B1, B6, B12, lidocaine, and betamethasone. The older patient had experienced recurrent episodes of weakness associated with low K^+^ levels. Both patients had muscle power scores of 0–1/5 in the upper and lower extremities, with areflexia. Laboratory results showed leukocytosis, with serum K^+^ levels of 1.6 and 1.8 mmol/L for the younger and older patients, respectively. TSH was within the normal range for both patients, and there was no evidence of renal K^+^ loss in urine biochemistry results. The patients received oral and IV KCl replacement in the ICU and were discharged with full recovery of muscle power (5/5). A notable case of potential interaction between glucocorticoids and another agent was reported by Shin et al. [22]. The authors described a case of betamethasone-induced hypokalemic paralysis in a patient who had also received tenofovir for hepatitis B virus infection. The authors suggested that the nephrotoxicity of tenofovir, particularly in the proximal tubules, may have exacerbated betamethasone-induced hypokalemia. The patient had serum K^+^ and phosphorus levels of 2.2 mEq/L and 1.1 mg/dL, respectively. The patient received tenofovir for two months but did not use it for the last four months. However, previous studies have noted the occurrence of Fanconi syndrome and hypokalemic paralysis after two to seven years of tenofovir use [26,27].

Although a large clinical study is lacking, it seems that, in addition to IM glucocorticoid injections, other administration routes, such as IV and epidural, can also induce hypokalemic paralysis in certain patients [13,[28], [29], [30]]. For example, two cases of epidural glucocorticoid-induced hypokalemic paralysis were recently reported [13,28]. The patients, aged 30 and 36, were males who received epidural steroid injections for low back pain. The first patient received an epidural injection of 10 mg dexamethasone, and after excluding other causes of hypokalemia, recovered and was discharged [28]. However, the second patient had a history of weight loss (30 lb) over the past six months and an enlarged thyroid gland on physical examination, prompting further thyroid function evaluations. The diagnosis of TPP was confirmed by a TSH level of 0.1 μIU/ml, along with elevated antibodies against thyroglobulin, thyroid peroxidase, and the TSH receptor [13]. Two other patients with thyrotoxicosis also developed hypokalemic paralysis following IV glucocorticoid infusions. The patients, aged 25 and 38, were men who experienced weakness in both upper and lower extremities following 2 g [29] and 500 mg [30] IV methylprednisolone. Notably, the first patient exhibited clear signs of thyrotoxicosis on physical examination, including exophthalmos and a diffusely enlarged thyroid. The second patient had a history of poorly controlled hyperthyroidism and was taking oral carbimazole (40 mg) and propranolol (40 mg). Both patients’ muscle strength fully recovered and were discharged after K^+^ replacement treatment.

Therefore, patients with thyrotoxic periodic paralysis (TPP) represent an important subgroup of those with glucocorticoid-induced hypokalemic paralysis. Lee et al. [5] reported two cases of thyrotoxic periodic paralysis (TPP) that were aggravated by glucocorticoids. The first patient, a 37-year-old man, presented with lower limb weakness after receiving a 10 mg dexamethasone injection. The second patient was a 35-year-old man with a history of Graves’ disease who presented with muscle weakness in both the lower and upper extremities after receiving a 5 mg dexamethasone injection. Thyroid function tests revealed thyrotoxicosis in both cases. The patients were fully recovered after K^+^ replacement and discharged with methimazole and propranolol. It is important to note that certain viral infections can cause alterations in thyroid function tests [[31], [32], [33]], and the interaction between thyroid hormones and glucocorticoid administration may exacerbate hypokalemia. However, in the case we reported here, the comprehensive history and physical examination were negative for thyrotoxicosis and TPP. For instance, our patient had no symptoms of weight loss, tremors, palpitations, skin or hair changes, heat intolerance, ophthalmopathy, or pretibial myxedema.

It is crucial to rule out serious neurological and toxicological conditions when evaluating a patient with acute-onset weakness. In our case, an emergent brain CT scan followed by a brain MRI were performed, both of which showed no lesions or ischemia. Moreover, the rapid improvement in muscle strength with K^+^ replacement therapy argues against other differential diagnoses, such as Guillain-Barré syndrome, myasthenia gravis, or spinal cord injury. Myopathy and rhabdomyolysis were ruled out due to normal CPK and LDH levels. Additionally, hypomagnesemia as a potential cause of hypokalemia was ruled out with a serum Mg level of 1.9 mg/dL, and the patient had no history of diuretic use, another common cause of hypokalemia. Some patients may experience hypokalemia following a GI illness [34,35]; nonetheless, a GI source of K^+^ loss was ruled out based on the patient's lack of history of diarrhea, vomiting, or laxative use.

An important differential diagnosis in adolescents with altered muscle strength and weakness is toxicity or adverse effects from substance abuse [36,37]. Certain drugs of abuse, particularly cocaine [38] and methamphetamine [39], have been associated with hypokalemia. However, urine toxicology tests and the physical examination ruled out common drugs of abuse in this patient. Several poisonings and medications can also be associated with hypokalemia. These agents include barium (e.g., from rodenticides) [40], insulin, chloroquine, beta-agonists, amphotericin B [24], and organophosphates [41]. The only drugs the patient had used were recent injections of dexamethasone and Neurobion. A thorough history, along with the absence of certain toxidromes, made other potential causes of drug-induced hypokalemia less likely.

## 4 Conclusion

In this case, we report a 17-year-old adolescent who developed acute hypokalemic paralysis following IM injection of dexamethasone and vitamin B1, B6, and B12 (Neurobion). Hypokalemia induced by glucocorticoids is a significant clinical concern, *via* activation of Na^+^/K^+^-ATPase, increased renal K^+^ excretion, and induction of hyperglycemia and hyperinsulinism. However, the underlying mechanisms remain not fully understood. Additionally, in patients with pernicious anemia, the administration of vitamin B12 could potentially lead to the synthesis of new erythrocytes, which shifts K^+^ ions into cells and may cause hypokalemia. Nonetheless, its role in non-anemic patients, in combination with other medications (e.g., glucocorticoids) and in patients with certain genetic predispositions, needs further research. Both glucocorticoids and B12 supplementation should be prescribed judiciously by clinicians to minimize potentially serious adverse effects. Future clinical and pathophysiological studies may explore the frequency, underlying mechanisms, and clinical significance of this potential interaction.

## CRediT authorship contribution statement

**Keivan Sahebi:** Writing – review & editing, Writing – original draft, Data curation. **Hassan Foroozand:** Writing – original draft. **Mohammad Bahmei:** Writing – original draft. **Raziee Taghizadeh:** Writing – review & editing, Supervision. **Samane Zare:** Writing – original draft. **Soroor Inaloo:** Writing – review & editing, Supervision.

## Patient perspective

From the patient's perspective, the experience was initially alarming due to the sudden and severe onset of paralysis. However, the early diagnosis and effective treatment that resulted in a complete recovery within a few days were reassuring. The clear communication from the doctors about the diagnosis, the absence of long-term complications, and patient education about preventive measures (e.g., avoiding high-carbohydrate meals) for the future added to their satisfaction. Overall, while the experience was initially distressing, the patient and his parents felt grateful for the high-quality medical care that led to a positive outcome and a full recovery without sequelae.

## Ethical compliance statement

The authors confirmed that the patient and his legal guardian were informed about the purpose and methodology of the study and voluntarily entered in the study. Informed written consent to participate and publish was obtained from the patient and his legal guardian (ethical code: IR.SUMS.MED.REC.1403.153).

## Data availability statement

The data are available from the corresponding authors on reasonable request.

## Funding sources

No specific funding was received for this work.

## Declaration of competing interest

The authors declare that they have no known competing financial interests or personal relationships that could have appeared to influence the work reported in this paper.
